# Immune Microenvironment and Genetic Signatures of End-Stage Renal Disease and Their Association with Sepsis: Insights from Public Transcriptomic Data and a Multicenter Clinical Cohort

**DOI:** 10.3390/biomedicines14040885

**Published:** 2026-04-13

**Authors:** Sheng-Huei Wang, Kuang-Yao Yang, Chau-Chyun Sheu, Biing-Ru Wu, Ming-Cheng Chan, Jia-Yih Feng, Chia-Min Chen, Yi-Cheng Shen, Wei-Hsuan Huang, Chung-Kan Peng, Shih-Ming Huang

**Affiliations:** 1 Institute of Medical Sciences, College of Medicine, National Defense Medical University, Taipei 114202, Taiwan; slidersinker@gmail.com; 2 Division of Pulmonary and Critical Care Medicine, Department of Internal Medicine, Tri-Service General Hospital, National Defense Medical University, Taipei 114202, Taiwan; 3 Department of Chest Medicine, Taipei Veterans General Hospital, Taipei 112201, Taiwan; 4 Institute of Emergency and Critical Care Medicine, School of Medicine, National Yang Ming Chiao Tung University, Taipei 11217, Taiwan; 5 Cancer and Immunology Research Center, National Yang Ming Chiao Tung University, Taipei 112304, Taiwan; 6 Division of Pulmonary and Critical Care Medicine, Department of Internal Medicine, Kaohsiung Medical University Hospital, Kaohsiung Medical University, Kaohsiung 807, Taiwan; 7 Department of Internal Medicine, School of Medicine, College of Medicine, Kaohsiung Medical University, Kaohsiung 807, Taiwan; 8 Division of Pulmonary and Critical Care Medicine, Department of Internal Medicine, China Medical University Hospital, Taichung 404332, Taiwan; 9 Ph.D. Program in Translational Medicine, National Chung Hsing University, Taichung 402, Taiwan; 10 Rong Hsing Research Center for Translational Medicine, National Chung Hsing University, Taichung 402, Taiwan; 11 Department of Critical Care Medicine, Taichung Veterans General Hospital, Taichung 407219, Taiwan; 12 Department of Post-Baccalaureate Medicine, College of Medicine, National Chung Hsing University, Taichung 402202, Taiwan; 13 Division of Infectious Diseases, Department of Internal Medicine, Taichung Veterans General Hospital, Taichung 407, Taiwan; 14 Institute of Biochemistry, College of Biomedical Sciences, National Defense Medical University, Taipei 114201, Taiwan

**Keywords:** end-stage renal disease, sepsis, carbapenem-resistant *Acinetobacter baumannii*, transcriptomics, bioinformatics

## Abstract

**Background**: End-stage renal disease (ESRD) is an immunocompromised state that confers a high risk of infection. We aimed to integrate bioinformatics analyses with a clinical cohort to explore the association between ESRD and sepsis. **Methods**: We retrieved transcriptomic data from the Gene Expression Omnibus and used computational tools, including Gene Set Enrichment Analysis, the eXtreme Gradient Boosting algorithm, and Mendelian randomization, to characterize gene expression changes, biological pathways, and genetic features in ESRD and sepsis. A multicenter retrospective cohort of patients with sepsis due to carbapenem-resistant *Acinetobacter baumannii* (CRAB) pneumonia in intensive care units (ICUs) was used to compare clinical presentation and outcomes between patients with and without ESRD. **Results**: Differential gene expression analysis showed widespread transcriptomic dysregulation in ESRD, and functional enrichment analysis revealed perturbations in immune signaling and vesicular transport pathways. Both the innate and adaptive immune systems appeared compromised, with marked depletion of lymphoid lineages in ESRD. An XGBoost machine-learning model derived from immune cell enrichment scores demonstrated a similar immune microenvironment in ESRD and sepsis. Mendelian randomization analysis supported an association between genetic variants predisposing to ESRD and an increased risk of sepsis, using genome-wide association study datasets. In the clinical cohort, patients with ESRD had significantly higher Sequential Organ Failure Assessment (SOFA) scores and in-hospital mortality than patients with normal renal function. **Conclusions**: ESRD shares similar immune microenvironmental features and genetic signatures with sepsis. These shared characteristics may contribute to the greater sepsis severity and poorer outcomes observed in patients with ESRD.

## 1. Introduction

Sepsis is a syndrome characterized by acute organ dysfunction due to a dysregulated host response to infection, and pneumonia is one of its leading etiologies, resulting in substantial morbidity and mortality [1,2,3]. Pneumonia can be classified as community-acquired pneumonia or nosocomial pneumonia, and the latter can be further subclassified into hospital-acquired pneumonia (HAP) and ventilator-associated pneumonia (VAP). The in-hospital mortality of nosocomial pneumonia is approximately 30–50%, and this high mortality is influenced by host immunity, comorbidities, disease severity at presentation, and causative pathogens [4,5]. Carbapenem-resistant *Acinetobacter baumannii* (CRAB) has been listed in the “critical” group in the updated World Health Organization bacterial priority pathogens list; CRAB pneumonia is associated with poor outcomes because of its virulence and the limited availability of effective antimicrobial agents in clinical practice [6,7]. CRAB infection can lead to more severe sepsis and catastrophic outcomes, particularly in critically ill patients in the ICU or in those with comorbidities such as ESRD [8].

ESRD is defined by the presence of uremic manifestations and the need for renal replacement therapy, including dialysis or kidney transplantation [9]. The global prevalence of ESRD is approximately 200–2000 per million population, whereas in Taiwan it exceeds 3000 per million population, largely due to diabetic nephropathy [10,11]. Patients with ESRD are immunocompromised because of dysfunction in both innate and adaptive immune responses involving neutrophils, macrophages, natural killer cells, dendritic cells, T cells, and B cells [12].

Prior studies have reported higher mortality in patients with ESRD than in those without ESRD when sepsis occurs [13,14]. Although this adverse outcome is plausibly attributable to the immunocompromised state, the relationship between the immune microenvironment and genetic signatures of ESRD and those of sepsis remains incompletely understood. Rather than re-reporting the previously described ESRD-versus-control transcriptomic differences, we used these public datasets as a basis for an integrative analysis to explore the biological and clinical links between ESRD and sepsis. In this study, we applied bioinformatics tools to characterize the immune transcriptome in ESRD compared with healthy controls, developed a machine-learning model based on immune cell enrichment scores to predict sepsis and applied it to ESRD cohorts, and used genome-wide association study (GWAS) data to explore the association between genetic variants linked to ESRD and sepsis. Finally, to translate these findings into clinical practice, we examined sepsis severity and outcomes among ICU patients with CRAB nosocomial pneumonia, stratified by renal function status (ESRD versus normal renal function) in a multicenter cohort in Taiwan.

## 2. Materials and Methods

### 2.1. Gene Expression Datasets and Preprocessing

All transcriptomic data used in this study were retrieved from the National Center for Biotechnology Information (NCBI) Gene Expression Omnibus (GEO) database. For the microarray dataset GSE37171 [15], raw CEL files were processed using the robust multi-array average (RMA) algorithm for background correction [16], followed by quantile normalization and probe-set summarization. For the RNA-sequencing (RNA-seq) datasets GSE97709 and GSE185263 [17,18], raw reads underwent quality control and were aligned to the human reference genome (GRCh38). A gene-level read count matrix was generated using featureCounts [19], and genes with low expression across most samples were removed.

### 2.2. Differential Gene Expression Analysis

Differential gene expression analysis between disease and control groups was performed using the edgeR package 4.6.3 in R/Bioconductor. edgeR models read counts with a negative binomial distribution to account for biological and technical variability [20]. The core steps included: (1) normalization for library size differences using the trimmed mean of M values (TMM) method [21], (2) estimation of dispersion parameters, including both a common dispersion across all genes and tagwise (gene-specific) dispersions, and (3) fitting of a generalized linear model to test for differential expression. An empirical Bayes procedure was used to moderate tagwise dispersions toward the common dispersion, improving inference, particularly in studies with small sample sizes. Genes were considered significantly differentially expressed if the Benjamini–Hochberg adjusted *p* value (false discovery rate [FDR]) was less than 0.05.

### 2.3. Functional Annotation and Pathway Enrichment Analysis

Functional annotation and pathway enrichment analyses of differentially expressed genes (DEGs) were performed using the ClueGO plugin in Cytoscape 3.10.4 [22]. The analysis was based on the Gene Ontology (GO) Biological Process database. Enrichment testing used a two-sided hypergeometric test, and *p* values were corrected for multiple testing using the Benjamini–Hochberg method. Functionally related GO terms were organized into a network, with nodes representing GO terms and edges indicating shared genes. The strength of the connection between terms was quantified by the kappa statistic, and a kappa score threshold of 0.4 was used. Tightly connected terms were clustered into functional groups, represented by the most significant term in each group. A detailed account of the analysis can be found in Fan et al. [23].

### 2.4. Datasets and Immune Feature Quantification

The sepsis gene expression dataset GSE185263 was used as the training cohort. An independent cohort comprising patients with ESRD, patients with uremia, and corresponding healthy controls served as the external prediction cohort. The xCell algorithm [24], implemented via the IOBR R package, was used to infer enrichment scores for 64 immune and stromal cell types from gene expression profiles. These enrichment scores served as input features for the machine-learning model. To integrate the training and external cohorts, the xCell enrichment score matrices were combined and batch effects [25] were corrected using the ComBat function from the sva R package. For more detailed information, readers are referred to the work of Chan et al. [26]. Because the ESRD/uremia and sepsis datasets were derived from different study populations, we used immune cell enrichment scores rather than raw expression values as a common feature space and applied batch correction before cross-cohort analysis.

### 2.5. XGBoost Model Development and Validation

We used the eXtreme Gradient Boosting (XGBoost) algorithm to construct a binary classification model to distinguish sepsis from healthy states [27]. The GSE185263 dataset was randomly partitioned into a training set (80%) and a testing set (20%). A grid search with 5-fold cross-validation was performed on the training set to identify optimal hyperparameters (eta = 0.01, max_depth = 4, colsample_bytree = 0.5, gamma = 0, min_child_weight = 1). The final model was trained on the full training set using these parameters. Model performance was evaluated on the independent testing set using the area under the receiver operating characteristic curve (AUC), precision, recall, F1 score, and the Matthews correlation coefficient (MCC). This concept has been extensively elaborated in the study conducted by Chen et al. [28].

### 2.6. Model Robustness Assessment and External Application

To assess the stability of feature importance, we performed an iterative procedure repeated 100 times. In each iteration, the data were randomly repartitioned (80:20), and the model was retrained to compute SHapley Additive exPlanations (SHAP) values. The importance ranking and frequency of each cell type were aggregated across all runs. The final trained model was then applied to the batch-corrected ESRD and uremia cohorts to estimate the predicted probability of sepsis for each sample.

### 2.7. Two-Sample Mendelian Randomization Analysis

We used a two-sample Mendelian randomization (MR) framework to assess the causal effect of genetic liability to ESRD on sepsis risk [29]. Genetic summary statistics were obtained from the IEU OpenGWAS database, with ESRD as the exposure (ID: ebi-a-GCST008031) and sepsis (critical care) as the outcome (ID: ieu-b-4982) [30]. Instrumental variables were single-nucleotide polymorphisms (SNPs) associated with ESRD at *p* < 5 × 10^−5^ and were clumped to ensure independence (linkage disequilibrium threshold r^2^ < 0.01 within a 10,000 kb window). All analyses were conducted in R using the TwoSampleMR package. The primary causal estimate was derived using the random-effects inverse-variance weighted (IVW) method [31]. Robustness was evaluated with several sensitivity analyses, including MR-Egger regression to assess directional pleiotropy [32], and the weighted median and weighted mode methods [33]. Results are presented as odds ratios (ORs) with 95% confidence intervals (CIs), representing the change in sepsis risk per unit increase in the log-odds of ESRD. Further methodological details can be found in Chou et al. [34].

### 2.8. Design of the Retrospective Cohort Study

We conducted a retrospective multicenter cohort study in five tertiary referral medical centers in Taiwan: Tri-Service General Hospital and Taipei Veterans General Hospital in northern Taiwan, Taichung Veterans General Hospital and China Medical University Hospital in central Taiwan, and Kaohsiung Medical University Hospital in southern Taiwan. These hospitals are major teaching centers with intensive care units and experience in the management of severe nosocomial pneumonia and multidrug-resistant infections. Patients with carbapenem-resistant Gram-negative bacteria (CR-GNB) pneumonia in the ICU between January and December 2016 were enrolled by our team focused on critical are and infection [35,36]. The inclusion criteria were: (1) diagnosis of HAP or VAP in the ICU, (2) pneumonia caused by CR-GNB isolated from qualified respiratory specimens, and (3) age ≥ 20 years. The exclusion criteria were: (1) lung cancer with obstructive pneumonitis, (2) pneumonia caused by pathogens other than CRAB, (3) no prescription of at least one parenteral antibiotic with in vitro activity against the index pathogen, and (4) a history of impaired renal function other than ESRD. After applying the inclusion and exclusion criteria, patients were classified into ESRD and normal renal function (NRF) groups, and their clinical presentation and mortality were compared. We focused on ESRD in the clinical cohort because ESRD is a well-defined clinical entity in retrospective multicenter data, whereas uremia is more heterogeneous and less consistently ascertainable from chart review.

### 2.9. Ethics Approval and Consent to Participate

This study was in accordance with the principles of the Declaration of Helsinki. The clinical cohort was approved by the institutional review boards of all participating hospitals, and informed consent was waived (Registration numbers: 2018-03-001CC, 1-107-05-054, CE18100A, CMUH107-REC3-052, KMUHIRB-E(I)-20180141).

### 2.10. Statistical Analysis

Student’s *t* test or the Mann–Whitney U test was used to compare continuous variables, and the chi-square test or Fisher’s exact test was used for categorical variables. Comparisons of predicted probabilities between groups (e.g., healthy controls vs. uremia) were performed using two-tailed Student’s *t* tests. All statistical analyses and visualizations were conducted in R. A *p* value < 0.05 was considered statistically significant.

## 3. Results

### 3.1. Widespread Transcriptomic Dysregulation Is a Hallmark of Uremia and ESRD

**Figure 1:**
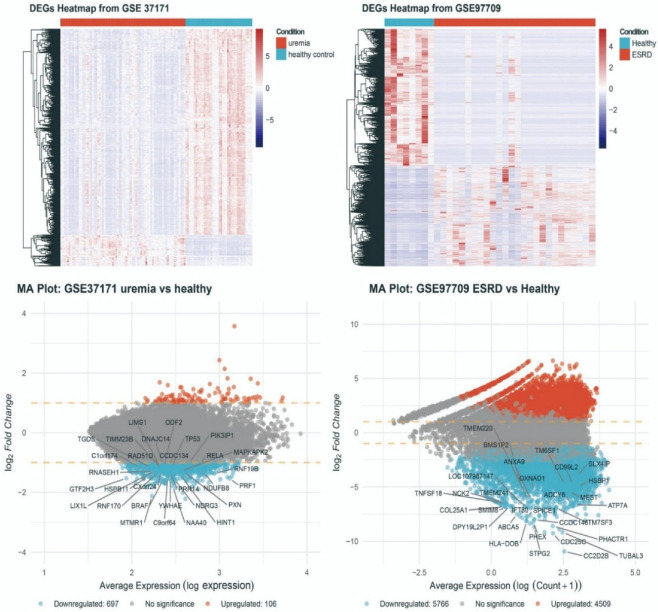
Widespread transcriptomic dysregulation in uremia and end-stage renal disease (ESRD). The upper panels show heatmaps of differentially expressed genes (DEGs) from GSE37171 (uremia vs. healthy controls) and GSE97709 (ESRD vs. healthy controls). Each row represents a DEG and each column represents a sample. Red indicates higher expression and blue/white indicates lower expression relative to the mean. The lower panels show MA plots for the corresponding datasets, plotting log2 fold change against average expression for each gene. Red dots represent significantly upregulated genes, blue dots represent significantly downregulated genes, and grey dots indicate genes without significant change, dashed lines indicate the threshold for differential expression (log2 fold change = ±1).

To investigate the systemic transcriptomic impact of renal failure, we first performed differential gene expression analysis on publicly available datasets from patients with uremia and ESRD. In GSE37171, comparing uremia with healthy controls, we identified 803 DEGs, including 106 upregulated and 697 downregulated genes (Figure 1). The heatmap analysis revealed clear clustering that separated uremia patients from healthy controls, indicating a distinct and consistent transcriptomic signature associated with the uremic state. The corresponding MA plot illustrates the relationship between average gene expression and log fold change, highlighting representative downregulated genes and upregulated genes.

In ESRD, transcriptomic perturbation was even more pronounced. In GSE97709, 10,275 DEGs were identified between ESRD patients and healthy controls, including 4509 upregulated and 5766 downregulated genes (Figure 1). This represents more than a 12-fold increase in the number of DEGs compared with uremia, suggesting that progression to ESRD is accompanied not only by a quantitative escalation in gene dysregulation but also by a progressive molecular dysfunction throughout the course of renal failure.

### 3.2. Functional Enrichment Analysis Reveals Perturbations in Immune Signaling and Vesicular Transport Pathways

**Figure 2:**
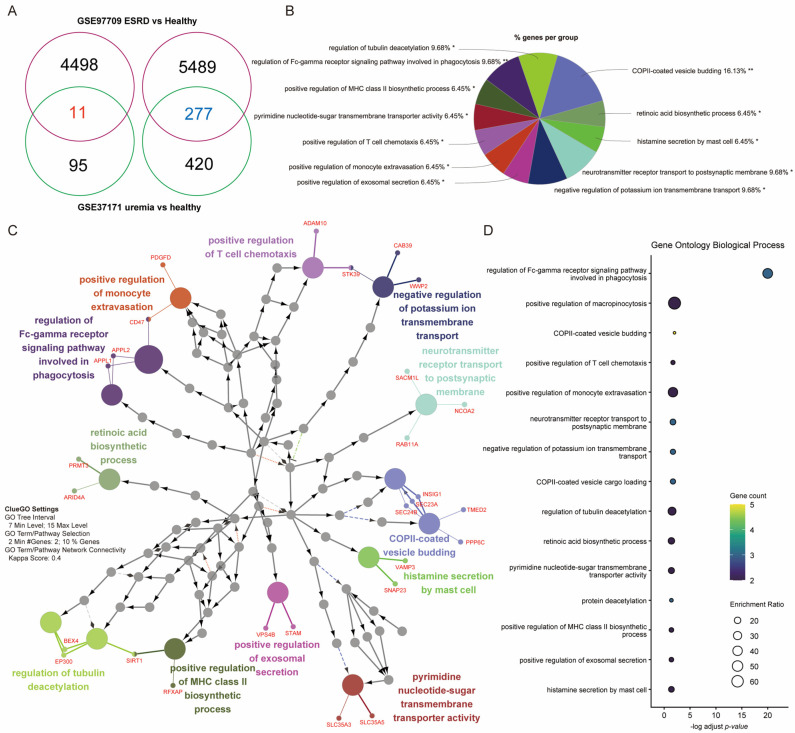
Functional enrichment analysis of common DEGs in uremia and ESRD. (**A**) Venn diagram showing the overlap of DEGs between GSE97709 (ESRD vs. healthy controls) and GSE37171 (uremia vs. healthy controls), identifying 277 common DEGs. (**B**) Network of enriched Gene Ontology (GO) Biological Process terms generated with ClueGO for the 277 common DEGs. Nodes represent GO terms, and edges connect terms that share a significant number of genes. (**C**) Pie chart depicting the percentage of genes from the input list that fall into the most significantly enriched functional groups. (**D**) Dot plot of the top enriched GO terms. The x-axis represents the log-transformed adjusted *p* value, the dot size indicates the enrichment ratio, and the color reflects the number of genes associated with each term. Asterisks indicate the significance level of enrichment: * *p* < 0.05; ** *p* < 0.01.

To elucidate the biological significance of the DEGs, we performed GO and pathway enrichment analyses on the 277 DEGs common to both the uremia (GSE37171) and ESRD (GSE97709) datasets (Figure 2A). Using ClueGO, we generated a network of functionally related biological processes (Figure 2B). Among the most significantly enriched pathways were those related to vesicular transport and immune function. The pie chart illustrates the distribution of genes across functional groups, with prominent categories including “coat protein complex II (COPII)-coated vesicle budding,” “regulation of Fc-gamma receptor signaling pathway involved in phagocytosis,” and “positive regulation of T cell chemotaxis” (Figure 2C). A dot plot highlights the statistical significance and gene counts for these enriched terms (Figure 2D).

### 3.3. Consistent Depletion of Lymphoid Lineages in Uremia and ESRD

**Figure 3:**
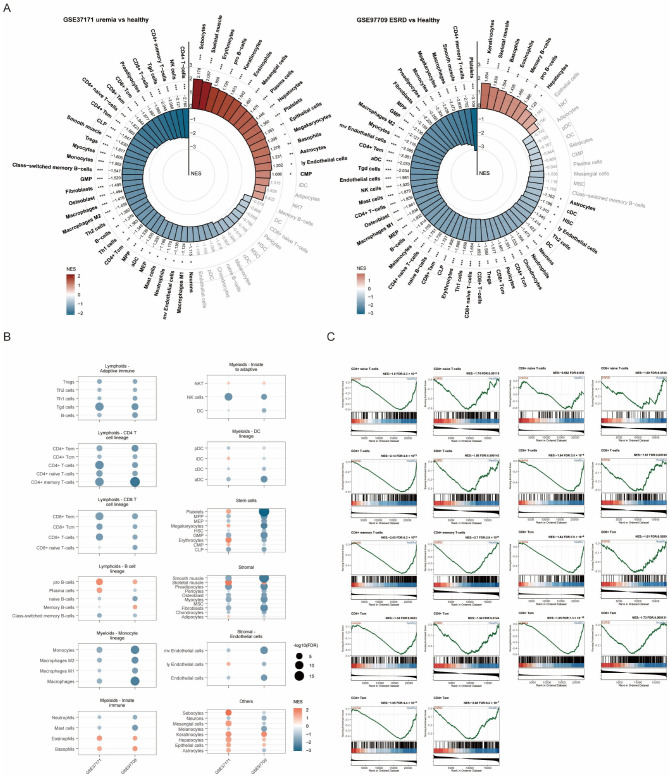
Enrichment analysis of immune and stromal cell infiltration in uremia and ESRD using xCell signatures. The figure illustrates the relative enrichment of 64 immune and stromal cell types inferred from bulk gene expression data. (**A**) Circular bar plots showing the normalized enrichment score (NES) for each cell type in GSE37171 (uremia vs. healthy controls, left) and GSE97709 (ESRD vs. healthy controls, right). Positive NES values (red) indicate enrichment, whereas negative values (blue) indicate depletion relative to healthy controls. The most significantly altered cell types are labeled. (**B**) Summary dot plot comparing NES values of key immune lineages (lymphoid, myeloid, stromal, etc.) across the two datasets, highlighting the shift in the immune landscape from uremia to ESRD. Dot color represents NES and dot size represents FDR significance. (**C**) Representative GSEA plots for specific T-cell signatures, such as CD4+ effector memory T cells (CD4+ Tem), showing the running enrichment score and the distribution of gene set members along the ranked gene list. Asterisks indicate statistical significance: * *p* < 0.05; ** *p* < 0.01; *** *p* < 0.001.

To define immune cell alterations in kidney failure, we analyzed two independent cohorts (GSE37171 and GSE97709) using gene set enrichment analysis (GSEA) with xCell signatures. The overall enrichment profiles, visualized in circular plots, show broad immunologic shifts compared with healthy controls (Figure 3A). The central finding, highlighted in the consolidated dot plot (Figure 3B), is a consistent and significant negative enrichment across most lymphoid cell lineages in both uremia and ESRD, indicating profound depletion of the adaptive immune system.

Specifically, key populations such as CD4+ T-cell subsets, CD8+ T cells, B cells, and plasma cells were significantly depleted. Innate lymphoid populations, including natural killer cells, also showed negative enrichment. To further validate these findings within the T-cell compartment, we examined the original GSEA enrichment plots for representative T-cell subsets (Figure 3C). The signatures for CD8+ central memory T cells, CD4+ T cells, Th1 cells, and Th2 cells all showed highly significant negative enrichment scores, indicating systematic downregulation of these T-cell gene signatures in advanced kidney disease and suggesting a state of T-cell lymphopenia or exhaustion.

### 3.4. Development of an Interpretable XGBoost Model to Predict Sepsis Using Immune Cell Signatures

**Figure 4:**
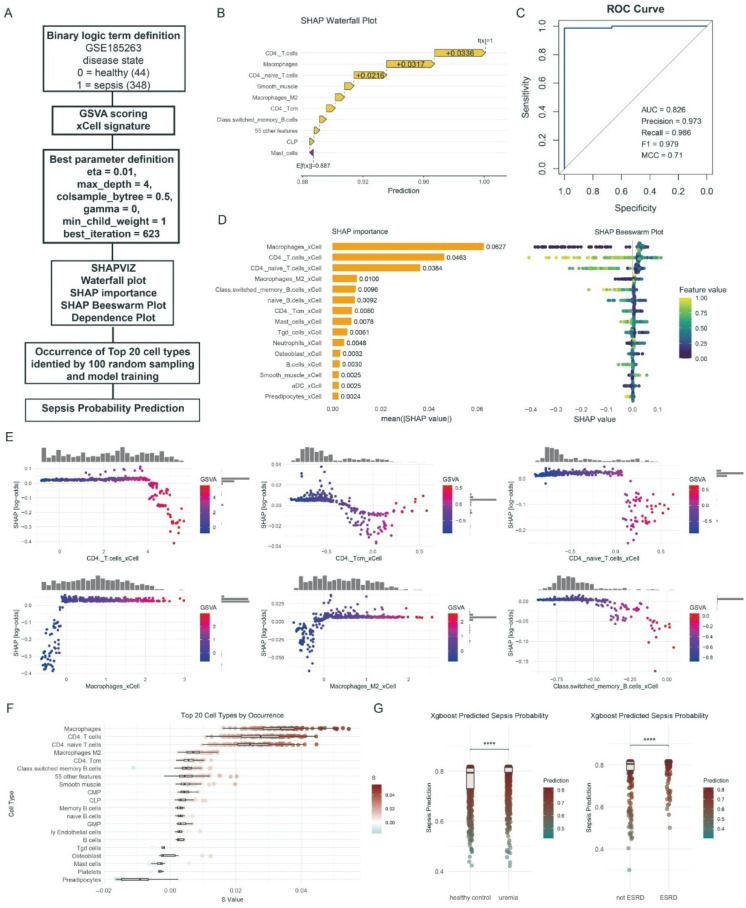
Development and interpretation of an XGBoost machine-learning model based on immune features. (**A**) Flowchart of the model development and analysis pipeline, including dataset definition, feature engineering with xCell, optimized parameter settings for the XGBoost model, and model interpretation with SHAP analysis. (**B**) SHAP waterfall plot for a representative sample, illustrating the magnitude and direction of each immune feature’s contribution to the predicted probability of sepsis. (**C**) Receiver-operating-characteristic (ROC) curve of the model, with AUC, precision, recall, F1 score, and MCC indicated. (**D**) Global feature importance plots. The bar plot (left) shows the mean absolute SHAP value for each feature. The beeswarm plot (right) shows the distribution, magnitude, and direction of SHAP values across samples. (**E**) SHAP dependence plots for key immune cell features, showing the relationship between the feature value (GSVA score) and its marginal contribution (SHAP value) to the model output. (**F**) Distribution of SHAP values for the 20 most frequently important cell types, derived from 100 iterations of model training. (**G**) Application of the trained XGBoost model to an independent cohort to predict sepsis probability in healthy controls versus uremia patients (left) and non-ESRD versus ESRD patients (right). Statistical significance was assessed with *t* tests; **** denotes *p* < 0.0001.

To investigate whether the immune remodeling observed in renal failure could be leveraged for sepsis prediction, we developed an XGBoost machine-learning model using the GSE185263 sepsis dataset (Figure 4A). The model was trained on immune cell enrichment scores inferred by xCell. It showed excellent performance for sepsis classification, with an AUC of 0.826, precision of 0.973, recall of 0.986, F1 score of 0.979, and MCC of 0.711 (Figure 4C), indicating high accuracy and reliability.

To interpret the model, we used SHAP analysis [37]. The SHAP importance plot identified macrophages (mean |SHAP value| = 0.0627), CD4+ T cells (0.0463), and naive CD4+ T cells (0.0364) as the three most influential features for sepsis prediction (Figure 4D). To confirm feature robustness, we repeated model training 100 times using random subsamples. Macrophages, CD4+ T cells, and naive CD4+ T cells consistently ranked among the most important predictors (Figure 4F).

SHAP waterfall and beeswarm plots further clarified the directional impact of key features (Figure 4B,D). Higher enrichment scores for CD4+ T cells, central memory CD4+ T cells, and class-switched memory B cells were associated with lower predicted probabilities of sepsis (negative SHAP values), whereas higher enrichment scores for M2 macrophages substantially increased the predicted probability of sepsis (positive SHAP values) (Figure 4E). When the trained model was applied to an independent cohort, patients with uremia had significantly higher predicted sepsis probabilities than healthy controls (*p* < 0.0001), and patients with ESRD had significantly higher predicted probabilities than non-ESRD controls (*p* < 0.0001) (Figure 4G). When the trained model was applied to the external cohorts, predicted sepsis probabilities were significantly higher in patients with uremia than in healthy controls and in patients with ESRD than in non-ESRD controls.

### 3.5. Mendelian Randomization Analysis Suggests a Possible Genetic Association Between ESRD and Sepsis

**Figure 5:**
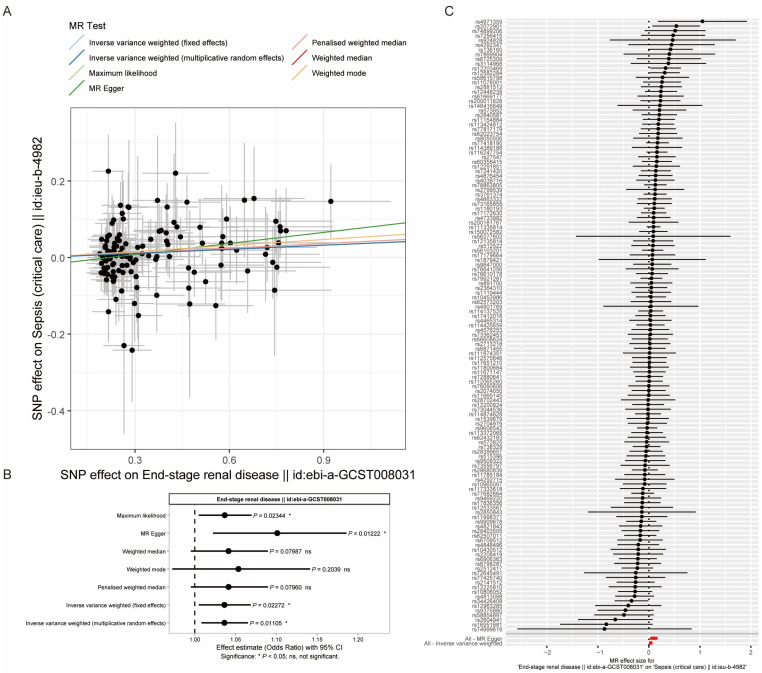
Two-sample Mendelian randomization (MR) analysis of the association between genetic liability to ESRD and sepsis. (**A**) Scatter plot illustrating the effect size of each instrumental SNP on ESRD (x-axis) versus its effect on sepsis (critical care) (y-axis). The slopes of the regression lines represent causal estimates from different MR methods. (**B**) Forest plot summarizing causal ORs and 95% CIs for the effect of ESRD on sepsis estimated by multiple MR methods. The vertical dashed line indicates the null value (OR = 1). (**C**) Forest plot displaying the causal estimate derived from each individual SNP. The overall IVW estimate is shown at the bottom in red, summarizing the combined causal effect.

To further explore whether genetic liability to ESRD may be associated with sepsis risk, we conducted a two-sample MR analysis (Figure 5). By using genetic variants as instrumental variables, this approach minimizes biases from confounding factors inherent in traditional observational studies. The results showed a modest but statistically significant association between genetic predisposition to ESRD (GWAS ID: ebi-a-GCST008031) and the risk of sepsis (critical care) (GWAS ID: ieu-b-4982).

Multiple MR methods yielded broadly consistent directions of effect, which provided supportive evidence for the observed association; however, the effect sizes were modest and should be interpreted with caution. Specifically, the primary Inverse-Variance Weighted (IVW) method (random effects) estimated an odds ratio (OR) of 1.04 (95% CI: 1.01–1.07; *p* = 1.11 × 10^−2^). This result was supported by maximum likelihood method (OR = 1.04; 95% CI: 1.01–1.07; *p* = 2.34 × 10^−2^), MR Egger (OR = 1.10, 95% CI: 1.02–1.19; *p* = 1.22 × 10^−2^) and IVW method (fixed effects) (OR = 1.04; 95% CI: 1.01–1.07; *p* = 2.27 × 10^−2^) (Figure 5B). The scatter plot (Figure 5A) and single-SNP forest plot (Figure 5C) visually confirm this positive association, demonstrating that genetic variants instrumenting for a higher risk of ESRD are correspondingly associated with a higher risk of sepsis.

### 3.6. Translation to Clinical Practice: Presentation and Outcomes of Sepsis Patients with CRAB Pneumonia in the ICU

**Figure 6:**
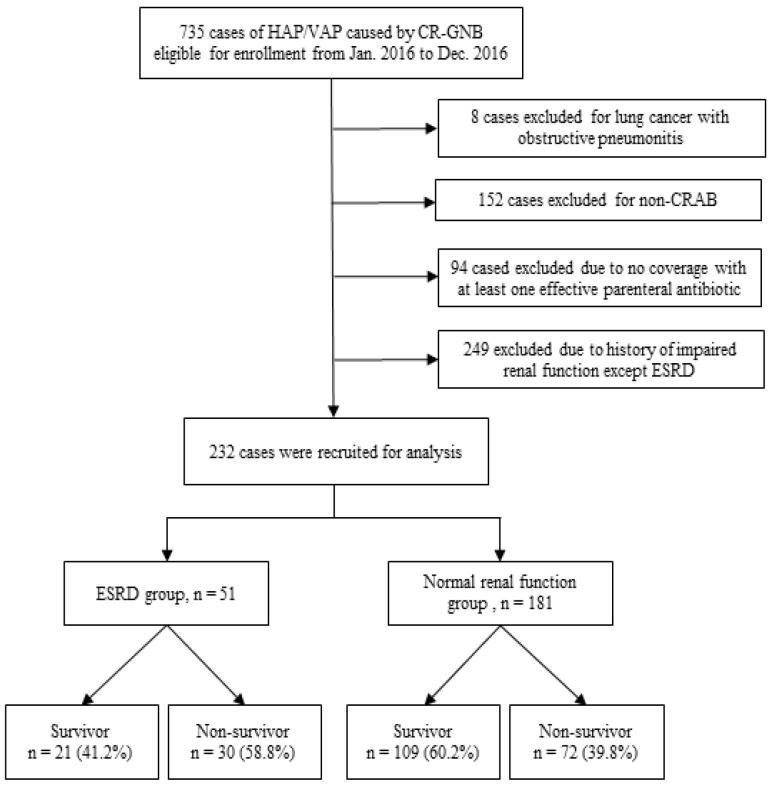
Flowchart of patient selection. Flowchart showing the selection of ICU patients with CRAB pneumonia and their classification into ESRD and normal renal function (NRF) groups after applying inclusion and exclusion criteria.

After applying the inclusion and exclusion criteria, 232 patients were included in the clinical cohort (Figure 6). As shown in Table 1, patients were classified into an ESRD group (*n* = 51) and an NRF group (*n* = 181). The two groups had similar baseline characteristics, including age, sex, body mass index (BMI), and pneumonia type (HAP vs. VAP). The prevalence of liver disease (15.7% vs. 5.0%; *p* = 0.015) and diabetes (58.8% vs. 33.1%; *p* = 0.001) was higher in the ESRD group than in the NRF group.

At pneumonia onset, the ESRD group had a higher SOFA score (10.0 vs. 7.2; *p* < 0.001) and higher plasma C-reactive protein (13.2 vs. 9.5 mg/dL; *p* = 0.004). In-hospital mortality was also significantly higher in the ESRD group than in the NRF group (58.8% vs. 39.8%; *p* = 0.017). The ESRD group had higher SOFA scores, higher CRP levels, and higher in-hospital mortality than the NRF group.

## 4. Discussion

In this study, we showed widespread transcriptomic dysregulation in ESRD using GEO datasets and identified perturbations in vesicular transport and immune-related pathways. We observed depletion of lymphoid lineages, particularly T-cell subsets, in ESRD. A machine-learning model and GWAS-based MR analysis revealed similarities and associations between the immune microenvironment and genetic signatures of ESRD and those of sepsis. Finally, in a multicenter retrospective ICU cohort of patients with sepsis due to CRAB pneumonia, patients with ESRD had higher SOFA scores and in-hospital mortality than patients with NRF.

Using two GEO cohorts and GSEA with xCell signatures, we identified significant negative enrichment across CD4+ T-cell subsets, CD8+ T cells, B cells, plasma cells, and NK cells in ESRD, consistent with previous observations that both innate and adaptive immune systems are compromised in chronic kidney disease, increasing the risk of infectious complications [38]. We further examined T-cell subsets and found that gene signatures for CD8+ central memory T cells, CD4+ T cells, Th1 cells, and Th2 cells were systematically downregulated, suggesting T-cell exhaustion in ESRD. The immune microenvironment in ESRD resembles that of sepsis, which is characterized by immune paralysis with impaired innate and adaptive immune responses and T-cell dysfunction due to apoptosis, lymphopenia, and functional alterations [39,40].

COPII-coated vesicle budding is crucial for trafficking proteins, lipids, and other cargo from the endoplasmic reticulum to the Golgi apparatus—an essential step for proper cellular function, including immune cell activity [41]. The co-enrichment of vesicular transport pathways (e.g., COPII vesicles, exosome secretion) and immune-related pathways (e.g., Fc-gamma receptor signaling, T-cell chemotaxis, major histocompatibility complex class II biosynthesis) observed in Figure 2 may reflect an underlying mechanistic link. Uremic cellular stress may disrupt protein and membrane trafficking, thereby impairing immune cell signaling, antigen presentation, and migration. Immune processes such as antigen presentation (via MHC molecules), phagocytosis (via Fc receptors), and chemotaxis (via chemokine receptors) depend on appropriate trafficking of membrane and secreted proteins [42,43]. Thus, dysregulated vesicular transport in ESRD is likely to have direct detrimental effects on immune function.

We developed a robust machine-learning model for sepsis prediction (Figure 4), in which macrophages, CD4+ T cells, and naive CD4+ T cells were the most important predictors. The model, built from immune cell enrichment scores, predicted higher sepsis probabilities in patients with ESRD than in controls. Sepsis is defined as infection complicated by organ dysfunction, rather than solely by immune dysregulation [44]. Therefore, the model predictions do not indicate that ESRD patients are in a septic state per se or that they will inevitably develop sepsis; rather, they suggest that the immune microenvironment in ESRD resembles that in sepsis and may predispose to more severe septic responses.

In the MR analysis (Figure 5), we used two GWAS datasets and confirmed a positive association between genetic predisposition to ESRD and sepsis risk. Given the principles of Mendelian randomization and the consistency of findings across IVW, MR-Egger, and weighted median approaches, our results support the existence of genetic variants that promote the pathway from ESRD to sepsis. This genetic signature complements the immunologic evidence and suggests that both immune dysfunction and genetic susceptibility may contribute to sepsis development in ESRD.

The bioinformatic and clinical analyses in this study were intended as two complementary parts of the same translational framework. The public transcriptomic and genetic datasets were used to explore whether ESRD shares biological features with sepsis, whereas the multicenter clinical cohort was used to examine the clinical relevance of these findings in a severe infection setting. In this way, the clinical cohort served not as mechanistic proof, but as a real-world context in which the potential impact of these shared biological features could be evaluated. In this study, the translational aspect referred to linking the bioinformatic findings to real-world clinical outcomes rather than direct bedside application. After identifying shared immune and genetic features between ESRD and sepsis, we examined a multicenter cohort of ICU patients with CRAB nosocomial pneumonia and found that patients with ESRD had greater illness severity and worse in-hospital outcomes. These findings suggest that ESRD may be recognized as a high-risk condition in severe infection and sepsis, supporting closer monitoring and more careful risk stratification in clinical practice.

This study has several limitations. First, the ESRD/uremia and sepsis datasets were obtained from different study populations and clinical settings. Although we reduced this issue by using xCell-derived immune features and ComBat batch correction, residual heterogeneity may still have influenced the cross-cohort comparisons. Second, although GSEA and xCell provide valuable insights, they are subject to inherent biases and estimation errors. Third, external validation of the machine-learning model with additional cohorts is needed to enhance its generalizability and robustness. Fourth, while we observed similar immune microenvironmental and genetic features in ESRD and sepsis, our analyses cannot fully delineate the mechanistic pathways from ESRD to sepsis; experimental and mechanistic studies will be required to clarify these pathways. Fifth, although the MR analysis showed statistically significant associations, the effect sizes were modest and the level of significance was borderline. Therefore, these findings should be interpreted cautiously and regarded as supportive rather than definitive evidence. Further targeted genetic, functional, and mechanistic studies are needed to validate this observation. Sixth, the clinical cohort was derived from patients enrolled in 2016, which ensured data completeness and consistency across centers but may limit direct generalizability to more recent clinical practice. Finally, the clinical cohort findings should be interpreted with caution. Because this was a retrospective analysis with a limited sample size, we were unable to perform a robust multivariable analysis to fully adjust for potential confounders. In particular, the ESRD group had a higher prevalence of diabetes and liver disease, which may have influenced the observed differences in severity and outcomes. Therefore, the clinical results should be regarded as unadjusted associations rather than evidence of an independent effect of ESRD.

## 5. Conclusions

In conclusion, we used bioinformatic approaches to characterize the immunocompromised state of ESRD and identified vesicular transport dysfunction as a potential contributing factor. A machine-learning model for sepsis and MR analysis revealed shared immune microenvironmental and genetic signatures between ESRD and sepsis. By integrating bioinformatics with clinical data, we demonstrated higher sepsis severity and worse outcomes in patients with ESRD than in those with NRF. The subtle and complex relationship between ESRD and sepsis warrants further investigation.

## Figures and Tables

**Table 1:** Baseline characteristics and outcomes of ICU patients with CRAB pneumonia stratified by renal function status.

	All (N = 232)	ESRD (N = 51)	Normal Renal Function (N = 181)	*p* Value
Age, M (SD)	71.4 (15.4)	70.3 (13.3)	71.8 (15.9)	0.276
Male, n (%)	145 (62.5)	31 (60.8)	114 (63.0)	0.870
BMI, M (SD)	22.5 (5.0)	22.2 (3.4)	22.7 (5.6)	0.286
Pneumonia Types, N (%)				0.999
VAP	179 (77.2)	39 (76.5)	140 (77.3)	
HAP	53 (22.8)	12 (23.5)	41 (22.7)	
Comorbidities, N (%)				
Malignancy	18 (7.8)	2 (3.9)	16 (8.8)	0.376
Stroke	32 (13.8)	8 (15.7)	24 (13.3)	0.649
Lung disease	34 (14.7)	10 (19.6)	24 (13.3)	0.267
Liver disease	17 (7.3)	8 (15.7)	9 (5.0)	0.015
Diabetes	90 (38.8)	30 (58.8)	60 (33.1)	0.001
Autoimmune disease	4 (1.7)	1 (2.0)	3 (1.7)	0.999
Disease Severity on Pneumonia Onset				
SOFA score, M (SD)	7.8 (4.0)	10.0 (3.2)	7.2 (3.9)	<0.001
Laboratory Results, M (SD)				
Plasma leukocyte (μL)	13,042.0 (9066.4)	15,168.4 (12,734.5)	12,442.8 (7668.2)	0.236
Plasma C-reactive protein (mg/dL)	10.5 (8.5)	13.2 (8.8)	9.5 (8.2)	0.004
Plasma albumin (g/dL)	2.7 (0.5)	2.7 (0.5)	2.7 (0.5)	0.933
In-Hospital Mortality, N (%)	102 (44)	30 (58.8)	72 (39.8)	0.017

M (SD) = Mean (Standard deviation).

## Data Availability

The transcriptomic datasets analyzed during the current study are available in the NCBI GEO database under accession numbers GSE37171, GSE97709, and GSE185263. Genetic summary statistics were obtained from the IEU OpenGWAS database (IDs: ebi-a-GCST008031 and ieu-b-4982).
